# Implementing and managing self-management skills training within primary care organisations: a national survey of the expert patients programme within its pilot phase

**DOI:** 10.1186/1748-5908-1-6

**Published:** 2006-02-23

**Authors:** Victoria Lee, Anne Kennedy, Anne Rogers

**Affiliations:** 1 National Primary Care Research and Development Centre (NPCRDC), 5^th ^Floor Williamson Building, The University of Manchester, Oxford Road, Manchester M13 9PL, UK

## Abstract

A key element of the United Kingdom (UK) health policy reform in relation to chronic disease management is the introduction of a national programme seeking to promote self-care from within the National Health Service (NHS). The mainstay of the Expert Patients Programme (EPP) is a six-week training course that provides the opportunity for anyone with a long-term condition to develop new skills to manage their condition better on a day-to-day basis. The course forms part of the NHS self-care support programme, is administered by Primary Care Trusts (PCTs) and delivered by people who have personal experience of living with a long-term condition.

The NHS' official Expert Patients Programme website presently states that, "Pilot EPP courses began at 26 NHS PCT sites across England in May 2002, and by May 2004 approximately 300 PCTs had either actively implemented pilot courses or had committed to joining. The majority of PCTs are now coming to the end of the pilot phase, with many implementing plans to make EPP sustainable for the long-term." The NHS website heralds the pilot "a success."

A national, postal survey of PCT EPP Leads was undertaken in order to examine both the evolvement of EPP during its pilot stage and future plans for the programme. A questionnaire was sent out to the 299 PCTs known to have committed to the EPP pilot, and an excellent 100% response rate was obtained over a 3-month period (April-July 2005). One marker of success of the Expert Patients Programme implementation is the actual running of courses by the Primary Care Trusts. This paper explores the extent to which the implementation of the pilot can indeed be viewed as a "success," primarily in terms of the number of courses run, and considers the extent to which PCTs have carried out all that they were committed to do. Findings suggest that the more time an EPP Lead dedicates to the Programme, the more likely it is that EPP has run successfully in the past, and the more likely it is that it will continue to run successfully in the future. Other factors indicating future EPP success include collaborating across PCTs to share co-ordinators, tutors, and funding.

## Introduction

The United Kingdom (UK) Labour government has introduced a wide programme of reform for the prevention and management of chronic conditions [1]. One of the key areas identified for action relates to the promotion of self-care. Self-care skills training has increasingly been seen as an effective strategy for improving the quality of life and health outcomes for people living with long-term conditions. Whilst the running of self-care skills training within the voluntary sector in the UK and Health Maintenance Organisations in the United States is not entirely new, in policy terms the top-down, state-sponsored national dissemination of self-care training represents a bold and novel development. The Expert Patients Programme (EPP) aimed to introduce a self-care skills training package embedded and integrated into the National Health Service (NHS) as part of a broader set of policy initiatives designed to address the needs of those suffering from long-term conditions [2].

The mainstay of the NHS EPP is a six-week generic training course that provides the opportunity for anyone with a long-term condition to develop new skills to manage their condition better on a day-to-day basis [3]. The course forms part of the NHS self-care support programme [4] and is delivered by people who have personal experience of living with a long-term condition (volunteer tutors with support from a small team of salaried trainers). Whilst it is the case that voluntary organizations have run support groups in the past and may deliver EPP in the future, during its pilot phase EPP has been administered by Primary Care Trusts (PCTs). These are free-standing statutory bodies responsible for delivering better health care and health improvements to their local area. PCTs have their own budgets and set their own priorities, although their activities are overseen by the Strategic Health Authority and national priorities set by the Department of Health. Thus, PCTs which currently provide and commission a wide range of primary care and community services are the organizations responsible for the implementation of EPP during its introductory phase throughout England. Within each PCT there is an individual who has overall responsibility for EPP – the EPP Lead – and it is to this person that the national postal survey was addressed and subsequently completed.

The process of embedding the EPP into the NHS has two components; firstly, running the lay-led self-care training courses for patients, and secondly, action-linking this to other practice and policies related to the management of long-term conditions already provided by the NHS and other agencies. During the pilot phase, PCTs received central funding to run four courses and to train two volunteer tutors. Expectations of central health policy makers in making such resources available imply that local agencies will be able to implement the programme as originally intended by the Department of Health. This paper explores the extent to which the implementation of the pilot by PCTs can be viewed as a "success," as the Department of Health have suggested it has been [5], and considers the extent to which PCTs have carried out all that they signed up to do.

## Findings

During the survey period (May 2002 to 1^st ^April 2005), which formed the final stage of a full process evaluation of EPP [6], a total of 1543 courses were run, with PCTs administering an average of five courses. Thirty PCTs (10%) had run more than eight courses, with 17 courses being the maximum number. The majority of PCTs (204, 68%) had run between four and eight courses. However, 65 PCTs (22%), a significant minority, had run less than the required four courses they had been funded to carry out. Further, of those 65 PCTs, 18 plan to run fewer than four courses in the present financial year. A total of 300 courses were cancelled during the survey period – 84 PCTs (29%) had to cancel two or more courses – with poor recruitment being by far the most commonly reported contributory factor (in 92% of cases), and appearing to be a universal problem.

Let us examine the 'PCT – course number' breakdown in a little more detail (Table 1). 'Champion' PCTs, meaning those that ran more than eight courses during the survey period, planned to run an average of eight courses in the 2005–06 financial year, with a mean budget of £14,000 assigned to EPP. They have an average of six tutors affiliated to the PCT (5 are active), and Leads would dedicate an average of 41% of their working week to the Programme. For the 204 PCTs that had run between four and eight courses, an average of five courses were planned with a mean budget of £9,000. They have an average of three active tutors, and Leads dedicate an average of 21% of their working week to the Programme. For those 65 PCTs that had not managed to complete the four-course requirement, an average of four courses have been planned with a mean budget of £8,000. They have an average of two active tutors, and Leads dedicate an average of 15% of their working week to EPP. Finally, for the 'lesser-achieving' 18 PCTs that had run fewer than four courses and planned to run fewer than four courses in 2005–06, they have a mean budget of just £2,000. They have an average of two tutors of which just one is active, and Leads dedicate an average of just 10% of their working week to the Programme.

**Table 1:** Past and Present EPP-related statistics with respect to 'PCT – course number' breakdown

	Mean Number of Courses Planned 2005–06	Mean Budget for EPP in 2005–06 (£)	Mean Number of Tutors/PCT in total	Mean Number of 'Active' Tutors	Mean % of Working Week Dedicated to EPP by Lead
30 PCTs ran > 8 courses	8	14,000	6	5	41
204 PCTs ran 4–8 courses	5	9,000	3	3	21
65 PCTs ran < 4 courses	4	8,000	2	2	15
18 PCTs ran < 4 and plan to run < 4 courses	< 4	2,000	2	1	10

One might logically assume a direct relationship between PCT size and the number of courses administered. Indeed, the bigger the PCT in terms of patient population (based on GMS census figures, 2004), the greater the number of courses run (r = 0.22, p < 0.001). Perhaps, not surprisingly, there are also direct correlations between PCT size and the number of courses planned to run in the 2005–06 financial year (r = 0.31, p < 0.001), budget assigned to EPP in this year (r = 0.22, p < 0.01), and the total number of PCT-affiliated tutors (r = 0.31, p < 0.001).

There is, however, **no **relationship between PCT size and the percentage time of a working week that an EPP Lead dedicates to Expert Patients Programme. Partial correlations, controlling for PCT size, show that the greater the percentage of time dedicated to EPP per working week by the Lead, the greater the number of courses that have been run (r = 0.32, p < 0.001), the fewer courses that had to be cancelled (r = - 0.17, p < 0.01), the more courses they plan to run in the 2005–06 financial year (r = 0.27, p < 0.001), the bigger the budget (r = 0.39, p < 0.001), the greater the number of tutors in total (r = 0.28, p < 0.001), and perhaps, more importantly, the greater the number of tutors actively engaged in delivering courses (r = 0.29, p < 0.001). In other words, the percentage of time that an EPP Lead commits to the Programme has a positive effect on all these variables, irrespective of PCT size.

## Discussion

There may be a number of alternative criteria by which the implementation of the EPP could be judged, such as evidence of EPP as a trigger for the development of user-initiated, independent support groups, or changes in health professionals' responses toward self-management, for example, which are explored in more detail in the Process Evaluation Report [6]. However, it should be noted that the purpose of this paper was to compare specific findings with the Department of Health's response to EPP *in its pilot phase*, and, as such, the success of the EPP is interpreted primarily in terms of the numbers of courses run and the anticipated numbers of future programmes.

The results of this national survey of PCT Leads examining the evolvement of EPP during its pilot phase, within England, suggest that irrespective of PCT size, the greater the percentage of time dedicated to EPP by the person leading this initiative – the more courses that have been run, the fewer the courses that have been cancelled, the more tutors affiliated with the PCT to deliver courses, and the more significant the planning for the future.

One limitation of this study is that we are unable to conclude causal relationships given the type of correlational analyses conducted and the data available. It would, however, be interesting to understand the nature of the time allocation (i.e. what tasks require greater time – recruitment, training of tutors, maintenance of tutors, organization of enrollment, running of courses, future planning etc.), and also which Lead tasks facilitate these factors [2]. Afterall, there is a need to focus the time of EPP Leads so that they can achieve maximum outcomes within the complex demands of PCT remits.

A total of 1305 courses have been planned for the 2005–06 financial year, with a total budget of £1,565,085 being assigned to EPP. So, it would seem that the more time an EPP Lead dedicates to EPP, the more likely it is that EPP has run successfully in the past, and the more likely it is that it will continue to run successfully in the future.

What does all this mean in terms of the future development of EPP by PCTs? Two-thirds of PCTs have EPP in their Local Development Plan which implies it is certain to be mainstreamed and allocated a budget. The current number of active tutors compounded by the difficulties of recruiting sufficient numbers to make a course viable, suggest that PCTs have the capacity to run a limited number of courses a year (involving about 40 to 50 people). Population size is clearly an issue. We found half the PCTs are actively collaborating with neighbouring PCTs to run courses. Pointers to future success of delivering the programme include appointing a dedicated EPP coordinator; and collaborating across PCTs to share coordinators, tutors and funding.

The current limit to most PCTs' capacity to devote to EPP suggests that the aspiration to view the EPP as a public health measure (in terms of population reach) is unlikely to be fulfilled in the short term, if PCTs are viewed as the primary means of delivering EPP. Although EPP in its pilot phase has been primarily PCT-led, voluntary organizations have historically run self-management support programs in the past and could possibly administer EPP in the future. Indeed, some "successful" (in terms of recruitment, courses run, and sustainability) PCTs are those who have begun to commission licensed voluntary organizations to recruit, administer, and effectively run the course.

Our results suggest that the EPP policy can be viewed as a "success," in so far as most PCTs ran a minimum number of training courses. However, our survey also illuminates the extent to which an 'implementation gap' [7] has arisen between the national aspirations for the EPP policy and local imperatives for delivery. The comparatively small number of courses that have been run means that the reach of the programme to those with long-term conditions has been limited and falls considerably short of what is expected of a public health policy, although the suitability of the programme for this population also may be an issue. As autonomous organisations, PCTs possess discretion in interpreting national policy, and this is clearly evident in the variations identified by the research reported here. In particular, we have seen that the success of the EPP relies to a large extent on the time and effort that PCT Leads working in local organisations dedicate to this particular policy, when faced with a number of competing priorities and policy preferences. Additionally, the limited capacity of PCTs to organize and recruit for training courses is compounded by having little direct access to patient groups. Thus, alternative and eclectic ways of delivering and accessing self-care training courses may be required. Recruiting directly from within primary and secondary care, and incorporating self-care training skills as part of routine disease management and care provided by professionals, could run alongside the current model which is dependent on lay leaders. This mixed approach may be the way forward, if EPP is to be sustained as it moves out of its pilot phase along a path of wider implementation and management.

## Competing interests

The author(s) declare that they have no competing interests.

## Authors' contributions

VL contributed substantially to the design, data collection, analysis and interpretation, and drafted the manuscript.

AK contributed to the design, analysis and interpretation of data, and revised the manuscript.

AR contributed to the design, analysis and interpretation of data, and revised the manuscript.

All authors read and approved the final manuscript.
